# Intralymphatic immunotherapy

**DOI:** 10.1186/s40413-014-0047-7

**Published:** 2015-03-07

**Authors:** Gabriela Senti, Thomas M Kündig

**Affiliations:** Clinical Trials Center, University Hospital Zurich, Raemistrasse 100/MOU2, CH-8091 Zurich, Switzerland; Department of Dermatology, University Hospital Zurich, Zurich, Switzerland

**Keywords:** Administration routes, Allergen immunotherapy, Intralympathic, Vaccination

## Abstract

Gold Standard allergen-specific immunotherapy is associated with low efficacy because it requires either many subcutaneous injections of allergen or even more numerous sublingual allergen administrations to achieve amelioration of symptoms. Intralymphatic vaccination can maximize immunogenicity and hence efficacy. We and others have demonstrated that as few as three low dose intralymphatic allergen administrations are sufficient to effectively alleviate symptoms. Results of recent prospective and controlled trials suggest that this strategy may be an effective form of allergen immunotherapy.

## Introduction

Specific immunotherapy (SIT) is the only disease modifying therapy for IgE-mediated allergic diseases. Subcutaneous immunotherapy is still considered the gold standard. One of the more recent developments is intralymphatic immunotherapy.

Frey and Wenk proved in 1957 [1] with a series of elegant skin flap experiments that antigens need to reach lymph nodes via afferent lymph vessels to induce a T-cell response. More recently experiments in spleenless (Hox11−/−) and alymphoplastic (aly/aly) mutant mice have confirmed the importance of secondary lymphoid organs, or neo-lymphoid aggregates [2], for elicting immune responses [3].

Early in lymphocyte development T- and B cell receptors are randomly rearranged resulting in T and B cells carrying a diverse repertoire of receptors. While this provides the ability of specific recognition of all possible antigens, it also requires antigens to be presented to approximately 10^7^ T- and B cells before eliciting an immune response. Therefore, only antigens that are washed into secondary lymphoid organs, where exposure to high numbers of T and B cells can occur, will generate an immune response. Antigens, however, that bypass secondary lymphoid organs have a reduced likelihood to encounter specific T or B cells, and are thus largely ignored. The phenomenon is termed the “geographic concept of immunogenicity” [4-6]. This concept remains valid although it may appear rather simplistic in the light of current understanding of immune regulation by dendritic cells and T cells. Being aware of the complexity of immune regulation we should none the less remember that the key trigger and regulator of the immune response is the antigen.

The lymph vessels role has evolved to drain pathogens into lymph nodes, thus enabling the immune system to generate an immune response at the earliest. Small particles of 20–200 nm size, i.e. the size of viruses, are quite efficiently drained in a free form from peripheral injection sites into lymph nodes. Usually, however, only a few percent of the injected particles reach the lymph nodes [7]. Larger particles in the size range 500–2000 nm are mostly carried into lymph nodes by DCs [7]. Non-particulate antigens, however, are much less efficiently transported into lymph nodes. Only a very small fraction, i.e. between 10^−3^ and 10^−6^, of the injected doses arrive there. Many of today’s vaccines and immunotherapeutic agents are non-particulate, therefore the injection directly into a lymph node should boost antigen presentation in the lymph node and thence improve the immune response.

## Review

As early as in 1977 a first review on intralymphatic vaccination was published [8]. In the early 1970s Juillard et al. used this method to enhance tumor cell based cancer vaccines in dogs. Ten years later, researchers were looking for the most efficient route of immunization for producing antibodies against purified proteins which were available in only very small amounts. In the 1980s reports were published of nanogram quantities of protein eliciting immune responses when injected into lymph nodes [9,10]. Thereafter in various fields where conventional routes of administration produced insufficient results or where maximizing the immune response was the goal, such as in cancer vaccines, intralymphatic vaccination was performed.

Intralympatic vaccination has been shown to improve the efficacy of various vaccines, e.g.

- BCG vaccines in dogs [8] and mice [11].
- DC-based cancer vaccines [12-18],
- Immunostimulating complexes (ISCOMS) [19],
- MHC class I binding peptide vaccines [20,21],
- Naked DNA vaccines [21-27],
- Protein based vaccines for immunization of macaques against SIV [28-34],
- Protein based vaccines in cows [35],
- Tumor cell-based cancer vaccines [4,8,36-40],
- Vaccines in cats against feline immunodeficiency virus using a protein based vaccine [41],

Moreover, lymph node targeting can also enhance the efficacy of adjuvants. Intralymphatic administration of the adjuvant CpG required 100 times lower doses of antigen compared with subcutaneous administration. Lower doses avoid undesired systemic adverse effects of the adjuvant [42]. This is in line with reports of enhanced efficacy of CpG and a better safety profile when targeting particles to lymph nodes [43,44].

**Figure 1:**
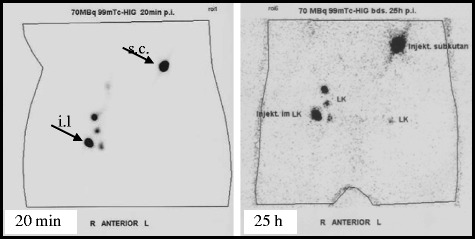
**Biodistribution after intralymphatic administration.** Biodistribution of 99mTc-labelled human IgG after intralymphatic (left abdominal side) and subcutaneous (right abdominal side) injections. Radio tracing was made by gamma-imaging 20 min (left panel) and 25 hours (right panel) after injection. Arrows indicate the site of injection (s.c., subcutaneous, i.l., intralymphatic).

Biodistribution studies in mice revealed that after direct lymph-node injection 100-fold higher antigen doses reached the lymph nodes than after subcutaneous injection in the drained area of a lymph node [45]. Intralymphatic and subcutaneous injections of radiotraced proteins in humans gave similar results. A ^99m^Tc-labeled protein was injected directly into a superficial inguinal lymph node on the right abdominal side. On the left side, the same dose was injected subcutaneously 10 cm above the inguinal lymph nodes. Figure 1 shows that only a small fraction of the subcutaneously administered protein had reached the lymph nodes after 4 hours, and that this fraction had not increased after 25 hours. In contrast, after intralymphatic injection the protein had drained into the deep subcutaneous lymph nodes and already after 20 minutes it was detected in a pelvic lymph node. Intralymphatic injection could efficiently pulse five lymph nodes with the full amount of the protein.

### Intralymphatic immunotherapy with allergen extracts

IgE-mediated allergies, such as allergic rhino-conjunctivitis and asthma today affect up to 35% of the population in westernized countries [46-49]. Subcutaneous allergen-specific immunotherapy (SIT) is the gold standard treatment, i.e. the administration of gradually increasing quantities of an allergen [50-52] over years. The immunotherapy confers long term symptom improvement [53-56], but the 30–80 visits of a physician over 3–5 years compromizes patient compliance. SIT is also associated with frequent allergic side effects and with a risk of anaphylaxis and death [57-59].

Allergen immunotherapy induces a phenotype shift in the T-cell response from Th2 to Th1 [60,61] and stimulates the generation of allergen-specific T-regulatory cells [60-62]. Serum titers of allergen-specific IgG antibodies, particularly IgG4, rise [63]. It is a matter of debate as to which of these immunological mediators is ultimately responsible for improving the allergic symptoms.

Intralymphatic administration of allergens to mice significantly enhanced the efficiency of immunization by inducing 10–20 times higher allergen-specific IgG2a antibody responses with as little as 0.1% of the allergen dose [45]. Intralymphatic injection of allergens also enhanced the secretion of IL-2, IL-4, IL-10 and IFN-γ compared to subcutaneous injection. This may indicate that intralymphatic administration does not polarize the response to the allergen, but overall generates a stronger Th1, Th2, and T-regulatory response [45].

Four separate clinical trials of the authors’ group have meanwhile demonstrated the feasibility, efficacy and safety of intralymphatic allergen immunotherapy. In the first clinical trial, eight patients allergic to bee-venom were given three low-dose injections of bee venom directly into their inguinal lymph nodes, whereas they would normally have received 70 subcutaneous injections. In this proof of concept trial seven of eight treated patients were protected against a subsequent bee sting challenge (Senti et al., manuscript in preparation). Similar results were achieved in a larger multi-center clinical trial with 66 bee venom-allergic patients (Senti et al., manuscript in preparation). In an other randomized controlled clinical trial, 165 patients with grass pollen-induced hay fever were administered either 54 subcutaneous injections with high dose pollen extract within three years or three low-dose intralymphatic injections over eight weeks. The three low-dose intralymphatic allergen injections reduced treatment time from three years to eight weeks and enhanced safety and efficacy of the treatment [64]. The results based on questionnaires and by combining patients treated with one of two allergens/seasons (grass and birch pollen) have been independently confirmed in a double-blind placebo-controlled trial using intralymphatic administration with the same dose, immunization regimen and grass pollen extract, and with tree pollen extract [65]. One trial with intralymphatic administration of grass pollen extract, however, only detected immunological alterations without clinical efficacy [66]. In that trial the time interval between injections was reduced to 2 weeks, whereas in the successful trials [64,65] the antigens were administered every 4 weeks. It is a well known fact of basic vaccine immunology that time intervals between injections of less than 4 weeks interferes with memory B cell formation and maturation of affinity [67,68]. Some authors, however, maintain that the time intervals argument is only valid for preventive vaccines, and that comparisons of low-power trials are strongly influenced by differences in endpoints and ways of assessment of clinical efficacy [69].

### Targeting intralymphatic vaccines to the MHC class II pathway

As intralymphatic vaccination brings the antigen directly to the lymph node DCs, the CD4+ T cell response may be enhanced by intracellular translocation sequences and sequences further targeting the antigen to the MHC class II pathway. Such allergy vaccines can be targeted to MHC class II molecules located in the endoplasmatic reticulum by fusing allergens to a tat-translocation peptide derived from HIV and to a part of the invariant chain. Several experimental studies have shown that such targeting not only bypasses the inefficient pinocytosis process but also the enzymatic degradation in phagolysosomes. Both can significantly enhance immunogenicity [45,70,71,72]. A first clinical trial has already proved this concept in a double blinded placebo-controlled setup [73].

### Intralymphatic immunotherapy is not painful

**Figure 2:**
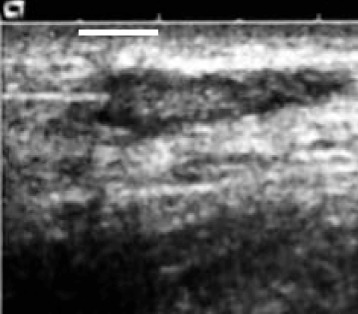
**Intralymphatic injection.** A sand blasted needle, being inserted into the lymph node from the right was used for better reflection and therefore visibility in the ultrasound. The dark, hypoechoic area represents the paracortex of the lymph node, which is approx. 15 mm long and 5 mm under the skin surface.

Subcutaneous lymph nodes are readily located by sonography since their paracortical area is hypoechoic (Figure 2). Injection into a superficial lymph node in the groin is usually performed in a few minutes and does not require great expertise in sonographic technique. What the patient feels during intralymphatic injection is solely the penetration of the skin, as lymph nodes carry few pain receptors. The pain of an intralymphatic injection thus is comparable with that of a subcutaneous injection. In the trials patients have rated intralymphatic injection less painful than venous puncture [64].

## Conclusions

Clinical trials indicate intralymphatic immunotherapy to be not only efficient and safe, but also more convenient for the patient, as well as associated with a lower risk of systemic adverse effects, including anaphylaxis and lethal consequences. With as little as 3 injections within 12 weeks, a relief of symptoms can be achieved that is comparable to that obtained with standard subcutaneous immunotherapy necessitating up to 100 injections over 3 to 5 years. As clinical evidence so far is available for grass pollen and bee venom, more clinical trials are required to assess the clinical usefulness of intralymphatic immunotherapy for other common allergens.

## Consent

Written informed consent was obtained from the patient for the publication of this report and any accompanying images.

## Abbreviations

BCG: Bacillus calmette-guérin
CD4+: Cluster of differentiation 4
CpG: Dinucleotide cytosine-phosphate-guanine
DC: Dendritic cell
HIV: Human immunodeficiency virus
IFNγ: Interferon gamma
IL-: Interleukin-
MHC: Major histocompatibility complex
SIT: Specific immunotherapy
SIV: Simian immunodeficiency virus
